# Deep learning predicts path-dependent plasticity

**DOI:** 10.1073/pnas.1911815116

**Published:** 2019-12-16

**Authors:** M. Mozaffar, R. Bostanabad, W. Chen, K. Ehmann, J. Cao, M. A. Bessa

**Affiliations:** ^a^Department of Mechanical Engineering, Northwestern University, Evanston, IL 60208;; ^b^Department of Mechanical and Aerospace Engineering, University of California, Irvine, CA 92617;; ^c^Department of Materials Science and Engineering, Delft University of Technology, 2628 CD Delft, The Netherlands

**Keywords:** deep learning, data-driven modeling, recurrent neural network, plasticity

## Abstract

Plasticity theory aims at describing the yield loci and work hardening of a material under general deformation states. Most of its complexity arises from the nontrivial dependence of the yield loci on the complete strain history of a material and its microstructure. This motivated 3 ingenious simplifications that underpinned a century of developments in this field: 1) yield criteria describing yield loci location; 2) associative or nonassociative flow rules defining the direction of plastic flow; and 3) effective stress–strain laws consistent with the plastic work equivalence principle. However, 2 key complications arise from these simplifications. First, finding equations that describe these 3 assumptions for materials with complex microstructures is not trivial. Second, yield surface evolution needs to be traced iteratively, i.e., through a return mapping algorithm. Here, we show that these assumptions are not needed in the context of sequence learning when using recurrent neural networks, diverting the above-mentioned complications. This work offers an alternative to currently established plasticity formulations by providing the foundations for finding history- and microstructure-dependent constitutive models through deep learning.

## Significance

We show that material plasticity can be precisely and efficiently predicted by deep-learning methods. This approach is fundamentally different from the century-old theory of continuum plasticity because it is not iteratively tracing the yield surface, neither does it require the notion of effective strain or stress at the macroscopic level. Instead, we use representative computer simulations of materials, including microstructure and constituents, load them along different deformation paths, and then learn the reversible, irreversible, and history-dependent phenomena directly from data. We demonstrate that complex phenomena such as distortional hardening can be predicted within 0.5% error. The generality of the methodology and widespread importance of plasticity in designing structures and materials make it useful to a myriad of fields.

History-dependent phenomena are common in natural and social systems (1). For example, the process of writing a new text starts by choosing the first word from all possible words, but then the words that follow depend on the past choices of words and their sequence. Plastic deformation of materials also depends on the history or path of deformation. If a bar is plastically deformed, the permanent strains that arise affect the subsequent deformation because the local stiffness changes. Therefore, predicting plastic behavior is nontrivial. We propose to address this challenge by using deep learning to find history- and microstructure-dependent plasticity models when abundant data of material behavior is available. This inverts the logic of continuum plasticity modeling where the goal is to predict inelastic deformation using minimal data from experimental testing. Deep-learning plasticity laws are shown to be computationally efficient and accurate, even when describing complex phenomena such as distortional hardening.

The remarkable success of continuum plasticity stems from reducing complex 3-dimensional (3D) inelastic phenomena to phenomenological laws defined by 1) a yield criterion, 2) a plastic flow normal to a plastic potential, and 3) an effective stress–strain law often defined from simple experiments (e.g., uniaxial tension). This achievement of describing plasticity for general stress states based on information from a limited amount of experiments should not be understated. The archetypal example is the von Mises model (2) with isotropic hardening, where metal plasticity is fully characterized (both yield surface and hardening) by a single uniaxial tensile test of the material. However, complexity of computational plasticity models tends to substantially grow as material behavior and plastic effects become more intricate when addressing phenomena such as the Bauschinger effect (3, 4), ratcheting (5, 6), anisotropy (7, 8), viscoplasticity (9), permanent softening (10, 11), and distortional hardening (12–14). These and other phenomena continue to be intensely investigated. For example, the past decade has introduced a shift from solutions based on the Armstrong–Frederick-type hardening (3) to models based on multiple yield surfaces (11) and distortional hardening (14). Still, the fundamental principle of reducing hardening of all 6 stress components to effective laws remains a central tenet to minimizing experimental testing, and the computational expense of finding iterative solutions for the plasticity equations remains an issue.

Modeling plasticity via machine learning requires a different perspective. Similarly to other fields where, for example, machine learning is helping to design new materials (15, 16) and to predict protein behavior (17), the key to learning constitutive models of materials is to generate data about material behavior. This has been achieved for nonlinear elastic constitutive laws where data are created by finite element analysis (FEA) of representative volume elements (RVEs) (18, 19) and, more recently, to determine property maps obtained from plasticity and fracture simulations of RVEs (19). As detailed in ref. 19, finding material models by machine learning is possible if the computational analyses of RVEs have high fidelity and sufficient efficiency to generate “enough” data for the supervised learning tasks. The required amount of data are problem-dependent, and generally more data are needed as the functional relation between the quantities of interest and the variables becomes more complex. Nevertheless, learning-plasticity behavior requires going one step further by considering sequence learning, a branch of deep learning, to incorporate history dependence and efficiently merge high-dimensional “temporal” strain paths and nontemporal geometry descriptors. Note that “temporal” strain paths here refer to snapshots of deformation, not rate dependency. We elaborate on this later.

## Theoretical Approach

Finding plasticity models can then follow the recently proposed 3-module data-driven framework (19) that integrates: 1) design of experiments to sample the input space; 2) computational analyses to create a database of outputs corresponding to each input configuration; and 3) machine learning to find the constitutive law that links inputs and outputs. As mentioned, here we focus on considering sequence learning for the third component, specifically recurrent neural networks (RNNs). Appropriate methodologies for design of experiments and computational analyses are also briefly described.

**Fig. 1.**
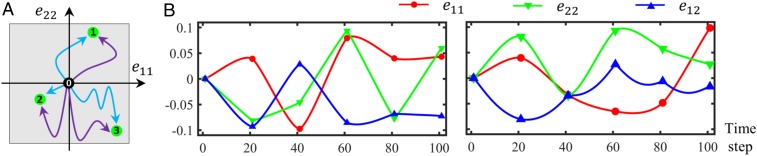
Sampling the temporally varying loads. (*A*) Three end states are marked in the strain space spanned by $e_{11}$ and $e_{22}$ ($e_{12}=0$ for clarity). For each end state, 2 deformation paths that connect it to the origin are illustrated. The gray area indicates the range of each strain component. (*B*) Two examples indicating the temporal evolution of the 3 strain components that, collectively, determine the deformation path to an end state. The markers on each path indicate the control points used in interpolation. Here, $n_{ts}=100$, $n_{cp}=6$, and the interpolator is a zero-mean GP with power exponential kernel. Paths in *B* are not related to *A*.

In general, finding constitutive laws by analyzing RVEs requires the definition of an input space with 3 types of variables: microstructural descriptors (e.g., volume fraction, inclusion geometries, etc.), material properties for each microstructural phase (e.g., elastic moduli, yield behavior, etc.), and loading conditions (e.g., average deformation, temperature, etc.). Past investigations and references therein (19) have shown that these temporally-fixed features are effectively sampled by Sobol sequence or Latin hypercube sampling when no prior knowledge is assumed (20). Sampling temporally varying features such as deformation path is more intricate as it involves generating a sequence of points (rather than a single point) for each sample path (Fig. 1*A*). To address this issue, we assume that any dynamic feature evolves to its end state in $n_{ts}$ steps of size Δt. From these steps, we then choose $n_{cp}$ equally spaced ones as control points and assign them random deformations which are uniformly drawn from the feature’s range. Finally, we realize a deformation path by connecting the strain component values of these control points via an interpolator (2 different examples in Fig. 1*B*). We have considered 2 different interpolators: Gaussian process (GP) and polynomial regression, see Fig. 1*B*. For multidimensional features such as strain, we generate paths along each dimension independently.

Following design of experiments, we analyze the resulting input database by implicit static FEA of heterogeneous RVEs under periodic boundary conditions (*Materials and Methods*). Therefore, finding the constitutive behavior of a heterogeneous material is possible by converting the average deformation applied to the RVE into a periodic boundary value problem and subsequently homogenizing the quantities of interest of that RVE (stresses and plastic energy) to save them in a database (19). Once the input space is sampled and the corresponding output database is created, an RNN is fitted to learn the plasticity-constitutive law by relating stresses and plastic energy to microstructure descriptors and loading conditions. RNNs describe plasticity as a map including the dependence on the sequence of deformation steps:

$${\bar{\sigma }}_{t}=f\left({\bar{e}}_{1:t},m,p,t\right),$$ [1]

where m and p describe the microstructure descriptors and the properties of the phases, respectively, ${\bar{e}}_{1:t}$ is the history of spatially averaged strains applied to the RVEs from the first to the current (t) deformation increment, and ${\bar{\sigma }}_{t}$ is the spatially averaged stress at the current deformation increment. Note that t could also represent “time” instead of “deformation” increments, i.e., it is a label for different snapshots. If we were interested in viscoplasticity, for example, then t would represent “time steps” or “frequency steps.”

RNNs are an extension of neural networks designed to handle sequential data, i.e., they can learn events happening along different time sequences (or deformation sequences, in this case) that can be captured with a different number of snapshots. RNNs use history-dependent hidden states $s_{t}\;\left(x_{t},s_{t-1}\right)$ to compute the outputs $o_{t}\left(x_{t},s_{t}\right)$. This enables them to carry information from previous inputs onto future predictions, where $x_{t}$ is the input feature at increment t. This unique feature of RNNs combined with the flexibility of their model architecture has proven to be greatly beneficial on tasks such as machine translation, natural language processing, and voice recognition, among others (21). Early formulations of RNNs suffer from a phenomenon known as vanishing/exploding gradients, first noticed in ref. 22, which hinders the backpropagation-based training process of the networks for long sequences. Long Short-Term Memory (LSTM) (23) was proposed to avoid vanishing gradients by using multiple data-gate mechanisms that control the flow of storing or forgetting information in hidden states and outputs. Gated Recurrent Unit (GRU) (24) uses a similar concept as LSTM while using a simplified formulation. Although LSTMs and GRUs have shown to have close performance in many cases (25), GRU’s formulation is less prone to overfitting and allows faster training due to the smaller number of trainable parameters.

**Fig. 2.**
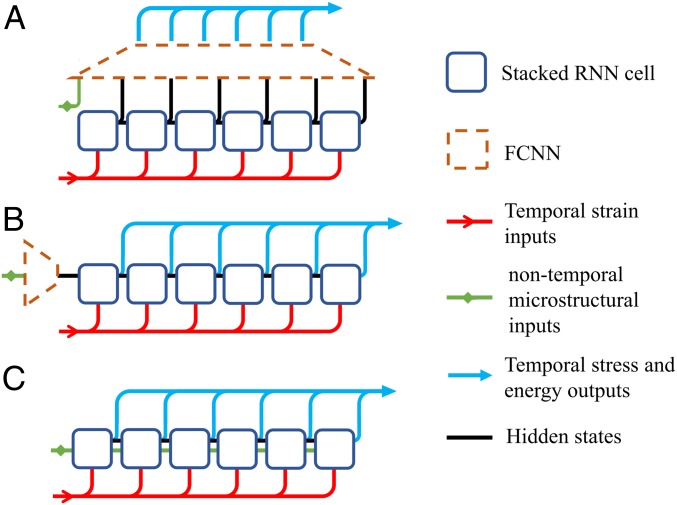
Variation of RNN architecture to encapsulate temporal and nontemporal inputs: postmixing nontemporal data through a dense network (*A*), configuring nontemporal data as initial hidden-state value through a dense network (*B*), or establishing a secondary nontemporal hidden state in GRU formulation (*C*).

A major challenge in predicting plasticity-constitutive laws for material systems is that the model should effectively correlate history-dependent (“temporal”) loading inputs with nontemporal RVE design features (e.g., volume fraction, fiber radius, or elastic moduli). Three variations of RNN architecture are considered to address this challenge, as depicted in Fig. 2 *A*–*C*. *SI Appendix* contains a comparative analysis between the 3 RNN architectures considered, where we demonstrate why we recommend the one shown in Fig. 2*C* for learning plasticity-constitutive models. In this architecture, a GRU formulation with a secondary hidden state is proposed to carry nontemporal inputs through the GRU cells, allowing nonlinear correlations between temporal and nontemporal input features while providing each GRU cell direct access to temporal inputs, nontemporal inputs, and history-dependent hidden states (Fig. 2*C*). Using this variation of GRU-formulation nontemporal features are protected from corruption by $r_{t}$, the reset gate. Although this approach adds additional weights and biases to the GRU, increasing model complexity and training time, it enables GRU cells to capture intricate interactions between input features throughout the entire temporal states. The altered formulation of a GRU unit (24) used in this work to combine temporal and nontemporal feature is as follows:

$$r_{t}=sig\left(W_{r}\cdot \left[h_{t-1},x_{t},h_{f}\right]+b_{r}\right),$$ [2]

$$z_{t}=sig\left(W_{z}\cdot \left[h_{t-1},x_{t},h_{f}\right]\;+b_{z}\right),$$ [3]

$${ĥ}_{t}=tanh\left(W\cdot \left[r_{t}\times h_{t-1},x_{t},h_{f}\right]\;+b\right),$$ [4]

$$o_{t}=h_{t}=\left(1-z_{t}\right)\times h_{t-1}+z_{t}\times {ĥ}_{t},$$ [5]

where sig is the sigmoid function. The reset gate ($r_{t}$) determines the combination of inputs ($x_{t}$), previous hidden states ($h_{t-1}$), and the secondary hidden state ($h_{f}$) to build a candidate hidden state (${ĥ}_{t}$). The update gate ($z_{t}$) controls the influence of the candidate hidden state to the unit output ($o_{t}$), which is also used as the new hidden state ($h_{t}$). Stacking multiple RNN units enables the model to predict higher-level nonlinearities and interactions between features.

Based on the architecture shown in Fig. 2*C*, we consider 2 examples to illustrate the capabilities of sequence learning in finding plasticity-constitutive laws. The first example focuses on a single RVE where there is significant distortional hardening. The second example considers a class of RVEs with different microstructures undergoing plasticity, demonstrating that our approach systematically incorporates microstructural information in plasticity-constitutive laws.

## Results

### Case 1: RVE with Distortional Hardening.

**Fig. 3.**
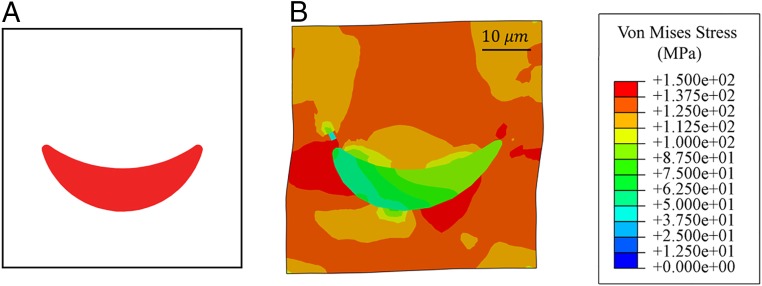
Undeformed configuration of RVE with curved ellipse (*A*) and von Mises stress contour of the deformed periodic RVE (*B*) in megapascals for illustrative case 1.

An illustrative example is devised by considering a periodic microstructure of a material composed of distorted elliptical fillers as shown in Fig. 3*A*. Without loss of generality, consider the matrix material to be an aluminum alloy (AA6061) described by a von Mises isotropic-hardening model, and the rubber fillers described by an Arruda–Boyce hyperelastic constitutive model (26). The material properties of the models and details of FEA are included in *SI Appendix*, Table S3. The combination of a ductile matrix and a hyperelastic filler with nonsymmetric geometry results in a compound elastoplastic behavior where the matrix deforms plastically while the fillers can store a significant amount of elastic energy. Although the constituents are isotropic, the filler geometry induces significant anisotropic behavior and distorts the yield surface obtained for the macroscopic heterogeneous material as it undergoes different deformation paths. The macroscopic constitutive behavior of the heterogeneous material results from relating the applied average strain components $e_{11}\left(t\right)$, $e_{22}\left(t\right)$, and $e_{12}\left(t\right)$ at deformation step t to the average stresses $\sigma_{11}\left(t\right)$, $\sigma_{22}\left(t\right)$, and $\sigma_{12}\left(t\right)$ and plastic energy $U_{p}\left(t\right)$. Note that each stress state of the RVE depends on the deformation path.

Once the RVE in Fig. 3*A* is simulated via FEA under 15,000 different deformation paths, a database with the average stresses and plastic energy for each deformation path is generated (100 deformation states per deformation path). Using this dataset (created in 2 wk using 48 cores), we train an RNN with an architecture illustrated in Fig. 2*C* whose parameters are trained on 80% of the database. We assess the predictive power and data sufficiency by the unseen 20% portion of data. We use a scaled mean absolute error (SMAE) metric to evaluate the results of the model and a second metric for the plastic energy, called scaled mean plastic energy decrease (SMPED), to quantify a possible decrease in plastic energy (the second law of thermodynamics does not permit a decrease). The designed model consists of 3 stacked layers of 500 RNN units followed by a single time-distributed dense layer, which corresponds to around 3 million trainable parameters. Finally, a leaky rectified linear unit (27) activation function is used to impose nonlinearity into the RNN. We trained the model for 200 epochs, which resulted in an SMAE of 0.00281 and 0.00355 for the training and test sets, respectively, and an SMPED of 0.000181 and 0.000186.

**Fig. 4.**
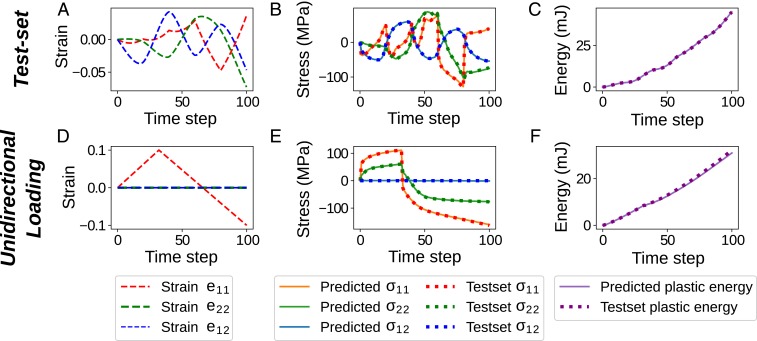
Evaluation results for the trained model in case 1. The top row demonstrates the applied average strains (*A*), the predicted and database average stresses (*B*), and the predicted and database plastic energies (*C*) for a test-set sample (unseen in the training process). The bottom row depicts the average strains (*D*), average stresses (*E*), and plastic energies (*F*) for the unidirectional loading test. Note the Poisson effect shown in *E* since $\sigma_{22}\neq 0$.

Fig. 4 presents results for 2 different validation deformation paths: one deformation path of the test set that was not used in training (Fig. 4 *A*–*C*); and a linear unidirectional loading and unloading deformation path for validation purposes that is not present in either the test or training sets (Fig. 4 *D*–*F*). As observed in Fig. 4 *B* and *C*, the RNN is predictive along deformation paths of the test set (unseen data) for both quantities of interest. Fig. 3*D* shows an unseen linear unidirectional strain path that stretches the RVE in the $e_{11}$ direction to 0.1 engineering strain and then in the opposite direction to −0.1 engineering strain, while $e_{22}$ and $e_{12}$ are kept at zero. Note that the training set does not include any linear strain path because it is constructed via Gaussian process regression of fluctuating paths, but Fig. 4 *E* and *F* demonstrates that our RNN model is still able to predict these average stress states and plastic energy. Fig. 4*E* also demonstrates that the RNN model predicts the Poisson effect ($\sigma_{22}\neq 0$), even though we did not impose this constraint in the strain paths because a valid material constitutive law needs to predict stresses for any given combination of the 3 strain components. *SI Appendix* provides details on the RNN architecture analysis (*SI Appendix*, Figs. S3, S4, and S5) with the corresponding error metrics used to assess the predictions.

**Fig. 5.**
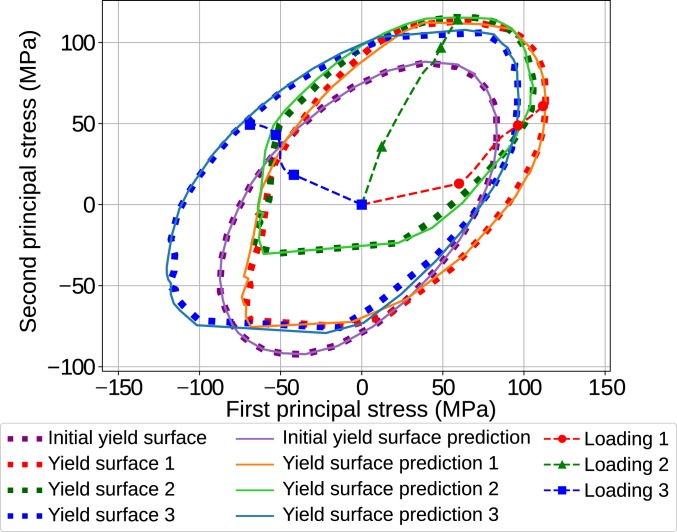
Yield-surface evolution under different deformation conditions and paths. FEA-based and RNN predicted yield surfaces are demonstrated in dotted lines and solid lines, respectively, at the end of 3 different deformation paths as compared to the original yield surface (purple).

We further explore the predictive capabilities of our model by evaluating the yield-surface evolution as the RVE experiences different loading conditions. Fig. 5 shows the yield surface at the onset of plasticity in purple, and the yield surface obtained at the end of 3 deformation paths. We define plastic deformation to start when the plastic energy increases by the threshold of 1 mJ. Alternatively, the average equivalent plastic strain of the matrix could be used. We construct the yield surface by loading the RVE from its current stress state to 40 deformation paths in different directions (*SI Appendix*, Fig. S6). The principal stresses of the RVE are calculated when the RVE is plastically deformed above the mentioned threshold and the stress state is stored in order to reconstruct the yield surface (details in *SI Appendix*). Each of the 4 yield surfaces shown in Fig. 5 includes the result obtained directly from FEA in dotted lines, the prediction from RNN in solid lines, and the applied deformation before yield surface construction with respective colors. The yield surface at the onset of plasticity (purple) resembles the elliptical shape of the von Mises yield surface, which is in-line with the matrix behavior. However, as seen at the end of the 3 deformation paths, the yield surface is distorted, shrinks/expands, and rotates for different deformation histories. Remarkably, the RNN can track the complete yielding behavior accurately, including the anisotropic and distortional yield behavior. Therefore, using sequence learning for finding plasticity laws of general RVEs is demonstrated to be possible, laying the foundations for a modeling route for plasticity that learns the compound correlation of yield surface and hardening laws without any explicit guide or definition of classical plasticity terms, such as effective plastic strain and effective stress.

### Case 2: Learning Plasticity for a Class of Composite RVEs.

**Fig. 6.**
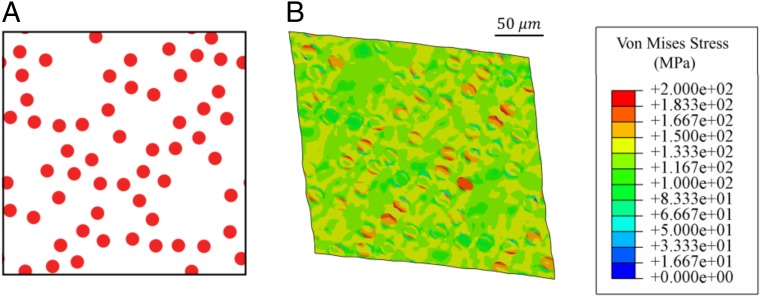
Undeformed configuration (*A*) and von Mises stress contour of a deformed sample of periodic RVE with distributed circular fillers (*B*) in megapascals for illustrative case 2.

A second case where a class of materials with periodic microstructure composed of different distributions of circular fibers is considered (Fig. 6), where each sample in the database varies in terms of their fiber volume (area) fraction v, fiber radius r, and distance between fibers c. We consider an epoxy matrix with combined isotropic and kinematic hardening, and carbon fibers with elastic behavior. Details of material properties are provided in *SI Appendix*, Table S4. We considered deformation paths with maximum strain of 8% in this case to explore the flexibility of the framework. These design choices give us a compound elastoplastic behavior for the RVE as the matrix deforms plastically while fibers can store a significant amount of elastic energy, whereas the behavior of the first case was mostly dominated by plastic deformation.

**Fig. 7.**
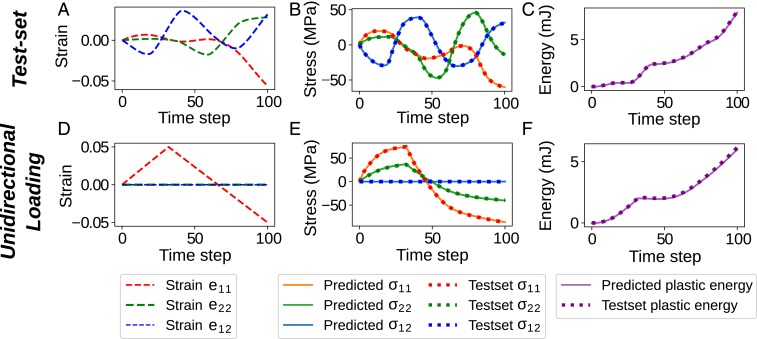
Evaluation results for the trained model in case 2 for 2 different RVEs under different loading conditions. The top row (*A–C*) corresponds to an RVE from the test set (unseen in the training process) that has a microstructure characterized by [v,r,c]≡[15%,6 μm,9 μm], shown in *SI Appendix*, Fig. S2 (RVE A). The bottom row (*D–F*) corresponds to a different RVE with microstructure characterized by [v,r,c]≡[6%,7 μm,6 μm], shown in *SI Appendix*, Fig. S2 (RVE B) and subjected to a unidirectional loading. The first column (*A* and *D*) shows the corresponding applied average strains; the second column (*B* and *E*) shows the average stresses predicted by the RNN model (solid line) compared to finite element analyses (dashed line); and the third column (*C* and *F*) shows the plastic energies predicted by RNN (solid line) and FEA (dashed line).

The inputs to the RNN model are the nontemporal microstructure descriptors, as well as the temporal deformation paths, and the outputs are temporal stresses and plastic energy over 100 increments for each RVE. Similar to the previous case, 2 validation tests are presented, and neither of which is used in the training process. A database with 8,000 samples is used in this case, 80% of which used for training. A model with architecture shown in Fig. 2*C* and similar configuration as the previous case is trained for 500 epochs resulting in 0.00132 and 0.00194 SMAE on the training set and test set correspondingly, while the model errors for the SMPED metric are 0.00179 and 0.00184, respectively. Fig. 7 *A*–*C* demonstrate the comparison of our model prediction with the ground truth FEA for a polynomial deformation path outside of the training samples. The results indicate that the model can accurately predict both stress and energy responses of the RVE. The results of linear unidirectional loading test (depicted in Fig. 7 *D*–*F*) show that the model is accurately predictive for stress and most regions of plastic energy. The small noise in the plastic-energy prediction is caused by the sharp change in deformation path. This was not observed when the deformation paths were sampled with GP regression due to its smoothness.

## Discussion

Recent progress in high-performance computing and reduced-order modeling are enabling fast and accurate predictions of the behavior of heterogeneous materials. This creates an ever-increasing number of large databases of material behavior that are suitable for analysis with machine learning. Here, we show that deep learning, and in particular sequence learning, becomes essential when dealing with history-dependent properties and constitutive behavior. We show that finding plasticity-constitutive laws of general materials and material classes is possible with unprecedented accuracy and efficiency. Our results indicate that the trained model can comfortably reach under 0.5% SMAE error, while being fast to evaluate (a fraction of a second, as discussed in *SI Appendix*) because there is no need for iterative-solution schemes such as Newton–Raphson, typical in classical plasticity. However, we note that deep learning strongly depends on the quality and quantity of the training data. For example, training data may be insufficient if the RVEs are computationally expensive or the RVEs may not accurately predict the behavior of real materials due to inaccurate local constitutive laws (19) and different sources of uncertainty (28). In such cases, the value of this approach deteriorates. Also note that data could be experimental, instead of computational. In that case, high-throughput experiments or open datasets that collect data from different sources should be considered, in order to have enough data.

We strongly believe that this is just the beginning of an exciting field in computational plasticity, as well as other fields in applied physics and engineering. Here, we focused on opening avenues for multiscale simulations where the macroscopic constitutive behavior reflects location-dependent heterogeneity of the microstructure. Yet, the approach is general and applicable to different time-dependent and history-dependent data. Future developments in reduced-order models enabling high-fidelity and high-throughput computational predictions of material behavior, together with the growing field of deep learning, will certainly create unprecedented conditions for discovering new materials and designing new structures undergoing extreme performance well beyond elastic limits.

## Materials and Methods

### Design of (Computational) Experiments.

We build an RVE database using a variant of the descriptor-based approach (29) as it establishes physically interpretable links between microstructural descriptors and material properties. First, we identify key microstructural descriptors that characterize the RVE and conduct design of experiments (DOE) on them. Then, we reconstruct the RVEs corresponding to the DOE points. Finally, we postprocess the generated RVEs to extract more microstructural descriptors that are not used in stage one. Since the material system in the second example is a fibrous composite where the fibers of an RVE are equally sized and randomly dispersed within the matrix, we choose the following 3 descriptors to characterize the morphology: fiber volume (or area) fraction (v), fiber radius (r), and minimum-allowable center-to-center distance between any 2 fibers (c). The first 2 descriptors are known to affect the material properties in fiber composites (19). The third descriptor is used to set a minimum distance between any 2 fibers to avoid overlaps, facilitate FEA, and partially control the spatial distribution of fibers within the matrix. Given the 3D input space of [v,r,c], we generate a DOE of size 8,000 where the range of each parameter is selected sufficiently large (*SI Appendix*, Table S1). For details about the reconstruction of a wide range of RVEs (case 2), see *SI Appendix*.

### Computational Analyses.

We simulate the behavior of the constructed RVEs under complex loading conditions using high-fidelity FEA. A MATLAB code developed by Bessa et al. (19) creates the scripts that interact with the finite element software ABAQUS to preprocess, execute, and postprocess all of the RVE simulations automatically using an implicit static solver. The analysis begins with generating the material and boundary condition files. The MATLAB script parses the geometry descriptors for each RVE in the database and generates python scripts, which create the geometry, mesh it with a predetermined mesh size (1.5 μm), and assign materials. The periodic boundary value problem follows by converting the applied average-strain components $e_{11}\left(t\right)$, $e_{22}\left(t\right)$, and $e_{12}\left(t\right)$ to displacement boundary conditions on edges of the RVEs. Next, the MATLAB script generates simulation files and executes ABAQUS to create the output simulations. Then, a final script executes the homogenization of the quantities of interest assuming separation of length scales (19), obtaining the average RVE stresses $\sigma_{11}\left(t\right)$, $\sigma_{22}\left(t\right)$, and $\sigma_{12}\left(t\right)$ as well as plastic energy $U_{p}\left(t\right)$. Note that the entire procedure is automatic and without human intervention.

### RNNs.

The proposed neural networks model based on the architecture demonstrated in Fig. 2*C* is developed using the Keras library (30). The model includes RNN cells to detect history-dependent features and combines with one or more time-distributed dense layers (30) to transform high-dimensional outputs of RNN cells into the desired 4 outputs (3 engineering stresses and plastic energy) of the RVEs over deformation increments. A cost function of the mean absolute error between the output values in the developed database and the predictions are defined and the training process is performed using Adam optimization method (31). The inputs (i.e., microstructure descriptors and deformation paths) and outputs (i.e., stresses and energies) of the database are normalized to a range between 0 and 1 to expedite the training process by reducing narrow valleys in trainable parameter space.

We use the SMAE metric to evaluate the results of the model, which is a fair error measure with the same dimensions as the original outputs. Although the plastic energy should not decrease over time according to the second law of thermodynamics, small decreases in the plastic energy can be seen even in the FEA results. To quantify if the plastic energy is predicted with an acceptable range of error, we define a second metric as the accumulated plastic-energy deviation averaged over test-set samples, which is named as SMPED. The developed model is trained on 80% of the database, while the rest is used as test set for validation. The train set SMAE decreases consistently as we increase the number of RNN layers, RNN units, or time-distributed layers due to the computational complexity of the models. However, using excessively complex configurations or adding extra time-distributed layers can adversely affect the accuracy of the model due to overfitting. *SI Appendix* contains further details.

## Supplementary Material

Supplementary File
