# Supplementary material for: Deep learning predicts path-dependent plasticity
Source: Proc Natl Acad Sci U S A. 2019 Dec 16;116(52):26414–20. doi: 10.1073/pnas.1911815116 (PMC6936363; doi:10.1073/pnas.1911815116)
Supplement: Supplementary File [file pnas.1911815116.sapp.pdf]

PNAS
www.pnas.org

Supplementary Information for

Foundations for Computational Plasticity with Deep Learning

M. Mozaffar, R. Bostanabad, W. Chen, K. Ehmann, J. Cao and M. A. Bessa

Corresponding Authors: M. A. Bessa and Jian Cao
E-mail: M.A.Bessa@tudelft.nl and jcao@northwestern.edu

This PDF file includes:

Supplementary text
Figs. S1 to S7
Tables S1 to S5
References for SI reference citations

www.pnas.org/cgi/doi/10.1073/pnas.1911815116

Supporting Information Text

Design of experiments

For case 2 considered in the main text, we reconstruct the RVE corresponding to the ith DOE point (i.e., given $[v_i, r_i, c_i]$) as follows. First we randomly place $n_i = \frac{v_i L^2}{100 * \pi * r_i^2}$ fibers of radius r_i in a square RVE of side length $L = 200\ \mu$m. Then, we iteratively perturb the fiber locations until their spatial distribution satisfies c_i. It is noted that some combinations of $[v, r, c]$ might correspond to infeasible RVEs or our iterative perturbation might stop before c_i is satisfied. The 3D input space of $[v, r, c]$ along with the feasible DOE points are visualized in Fig. S1 where it can be observed that some regions of the $[v, r, c]$ space do not correspond to realizable RVEs. Fig. S2 also shows four sample RVEs for easier interpretation of microstructural differences. Note that the triplets $[v, r, c]$ cannot uniquely characterize a microstructure with randomly dispersed equally-sized fibers (1). Hence, we post-process the reconstructed RVEs to extract four more morphological features that quantify the spatial distribution of fibers. These features are the minimum, maximum, mean, and standard deviation of nearest neighbor distances across the fibers: $nn = [nn_1, nn_2, nn_3, nn_4]$. That is, for the ith RVE, we calculate the nearest neighbor of all the fibers (center-to-center distance) and then calculate the above-mentioned statistics. These seven non-temporal features (along with the deformation path) are employed in our deep learning task as inputs. The deformation paths are sampled as described in the main text, such that the stress response is learned by training the RNN on a wide range of RVEs and deformation scenarios.

In summary, for case 2, Fig. S1 shows 5,000 sampling points obtained with Sobol sequence within the 3D space defined by the three microstructural descriptors of the RVEs: volume fraction v (in percentage), fiber radius r (in μm), and mean distance between fibers c (in μm). Fig. S2 shows four different samples of RVEs with different microstructure descriptors (four of the 5,000 points in Fig. S1).

RNN architecture analysis

The three RNN architectures introduced in Fig. 2 to combine temporal and non-temporal features are extensively tested and their training results are presented in Fig. S3 and Table S2 after 500 epochs of training on case 2 database.

Fig. 2A considers an architecture where non-temporal features are merged with temporal RNN outputs through fully connected neural network (FCNN) layers that form a hybrid deep learning architecture. While this approach is plausible for applications with fixed output length at the final time-step (i.e. deformation increment), its structure does not provide a natural fit for constitutive law discovery of material systems as it restrains the temporal prediction of the model to a fixed length and offers limited correlation between temporal and non-temporal features. Therefore, the hybrid architecture, which combines temporal GRU outputs with non-temporal FCNN features (Fig. 2A) cannot achieve accurate prediction on the training set and suffers from overfitting.

The second architecture shown in Fig. 2B has non-temporal features integrated into the RNN formulation as the initial value for hidden states. As the dimensionality of non-temporal inputs and hidden states are often different, a dense network can be used to perform this mapping. Although this approach has shown promising results for image processing tasks (2, 3), it is not the most effective architecture for constitute laws because all information in the hidden states are subject to change as they pass through GRU cells. That is, non-temporal inputs can get corrupted with other hidden features which makes it excessively difficult for GRU cells to access them at downstream time steps. Hence, this architecture with hidden state initialization performs moderately.

Finally, observing Fig. S3 and Table S2, we conclude that the proposed architecture with a secondary hidden state (Fig. 2C) achieves significantly better accuracy consistently across different epochs and metrics.

RNN hyperparameter tests

The hyperparameters and configurations of the presented RNN models are studied and optimized in this work. This analysis includes but is not limited to activation functions, optimization algorithms, cost functions, dropout layers, normalization process, and addition of time-series dense layers. Fig. S4A depicts the results achieved by varying number of neurons in each GRU cells. It can be seen that 100 neurons cannot provide enough computational complexity to the model. While the model with 1000 neurons results in lower SAME on training set compared to the model with 500 neurons, the models perform closely on the test set. Considering that the model with 1000 neurons requires more computational resources and training time and overfits on the training set, RNNs with 500 neurons are used in this work. Similarly, Fig. S4B suggests that a model with 3 layers of stacked GRU layers achieves the best result when compared to models with 1 or 5 layers.

Performance of proposed RNN architecture

We analyze the performance of the model with different sizes of training set to study the required database for achieving certain error metrics, which is demonstrated in Fig. S5. As we increase the size of the training set, the model with 3 layers of 500 neurons performs better in both training set and test set; however, larger databases lead to an expected improvement of performance. Ultimately, the require size of database is dictated by the complexity of the behavior of the RVE and the required accuracy. In this work, we demonstrate that one can achieve predictive deep learning models for advanced plasticity behavior with databases that are computationally (or experimentally) built in a feasible time frame.

Note that once trained, our data-driven constitutive model performs far faster than the finite element method. As an example, the developed data-driven model predicts the behavior of one RVE in the second case study in 0.108 seconds on a Nvidia Titan black GPU while it takes 7.48 minutes on four cores of Intel Xeon CPU E5-2687 for the finite element method to complete the simulation. While the exact number highly depends on the hardware and simulated physics, it can be confidently stated that the data-driven approach offers orders of magnitude faster evaluation. This has important implications on multi-scale simulations where the constitutive laws at each point of the macro-scale material can be given by RNN models, instead of expensive RVE analyses. Furthermore, we note that the two approaches scale differently, given the type of hardware they require and application. For instance, calculating the response of 100 different RVE cases via the data-driven approach using the same hardware takes only 0.547 seconds, which is due to the batch processing capability of GPUs. Finite element methods, on the other hand, scale by distributing sub-domains over multiple CPUs to obtain performance gains through parallel computing. These gains often saturate due to the communication overhead between processing units.

Yield surface construction and microstructural influence

The yield surfaces presented in Fig. 5 are constructed by applying 40 linear strain paths, which are uniformly distributed in strain space starting from the initial strain condition. To construct the original yield surface (Fig. S6A), strain paths start from the unloaded condition and experience elastic and plastic deformation in different directions. We record the stress state in which each linear path exceeds a plastic energy threshold of 1 mJ, which constructs the yield surface. The plastic energy is defined as the integral of stress times plastic strain over volume and over the deformation path: $\int_0^\tau \int_V \boldsymbol{\sigma} \cdot \boldsymbol{\varepsilon}^p \; dV d\tau$. The yield surface of an RVE after it undergoes a certain loading (Fig. S6B) is constructed by initially applying the main load (blue solid line in Fig. S6B) for all 40 linear strain paths and then loading the RVE in different directions until we detect the stress state where they reach the plastic energy threshold. Note that although all the applied loadings for yield surface constructions are linear and uniform in strain space, the stress responses are neither linear nor uniform which is due to the plasticity of the RVE.

In addition, we also illustrate the influence of the microstructure on the response of the material, as shown in Fig. S7, by considering the same loading condition applied to two different RVEs (C and D in Fig. S2). This clarifies the non-trivial relationship of microstructure and plastic response.

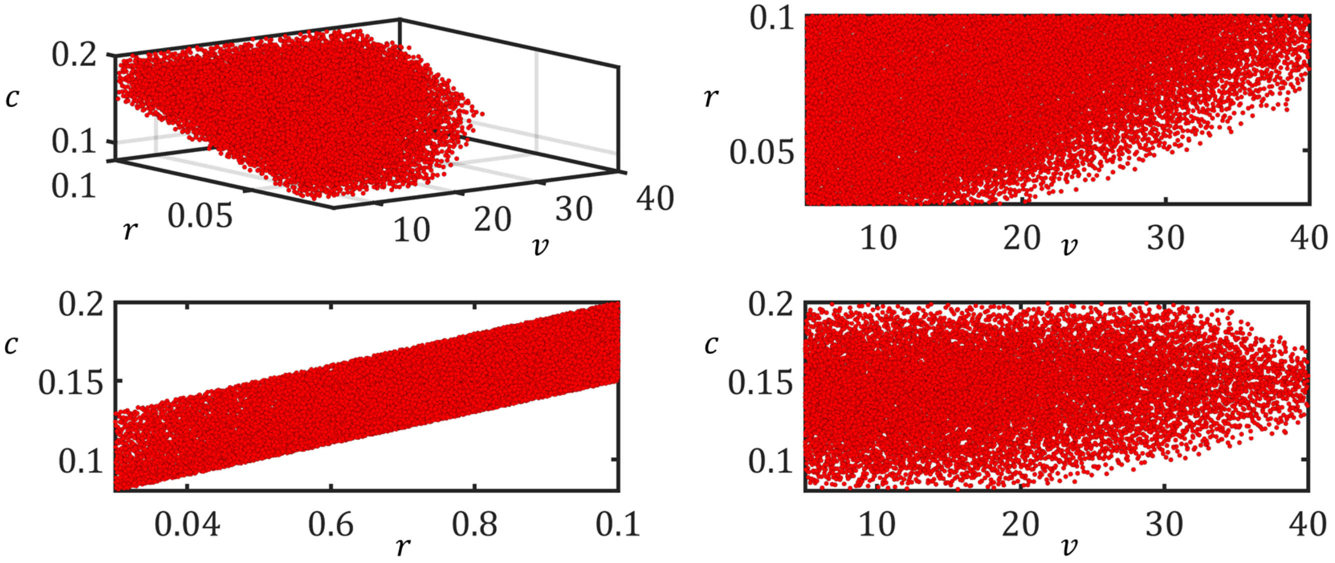

Fig. S1. Design of experiments with 5,000 points in the 3D space of $[v, r, c]$. v is in percent while r and c are in μm.

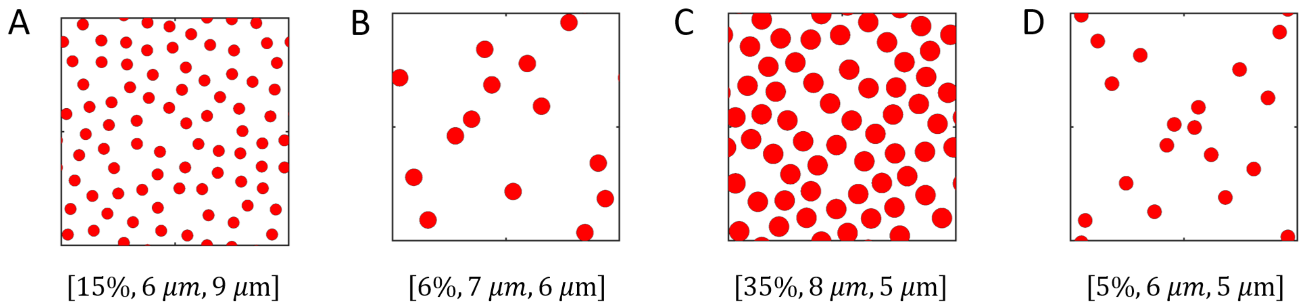

Fig. S2. Four sample RVEs. Side lengths are all $200\ \mu$m and the triplet below each RVE corresponds to $[v, r, c]$. v is in percent while r and c are in μm.

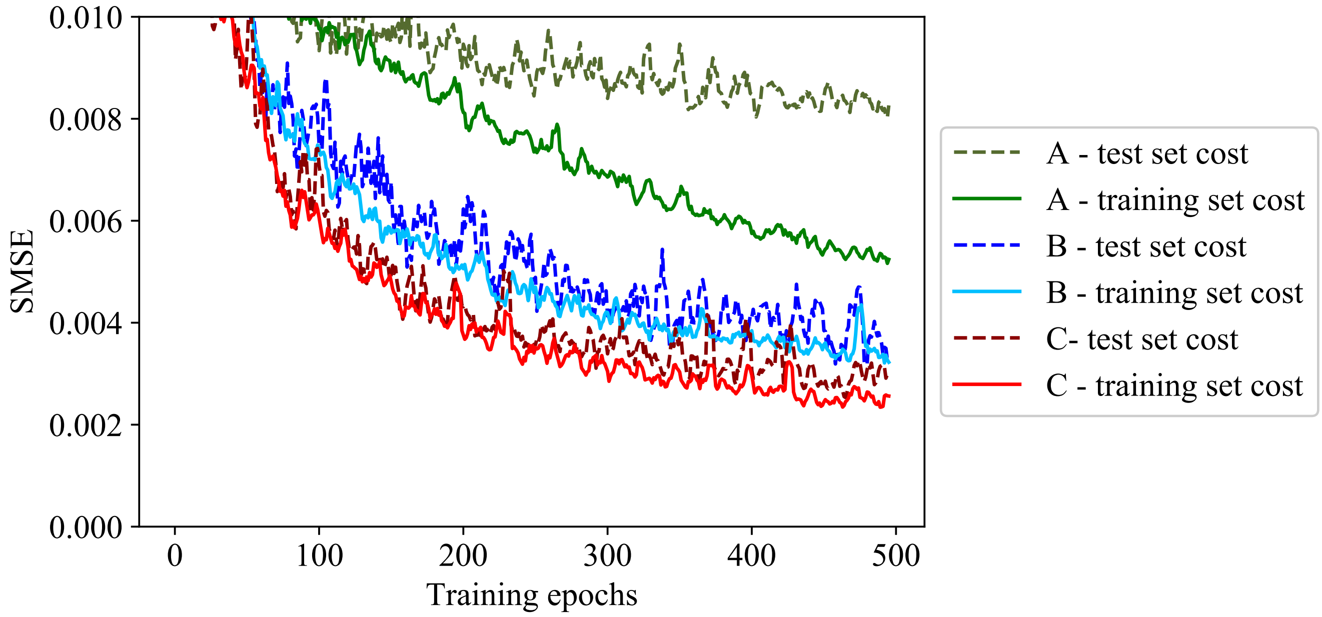

Fig. S3. Cost function evolution as a function of training epochs for three different RNN architectures.

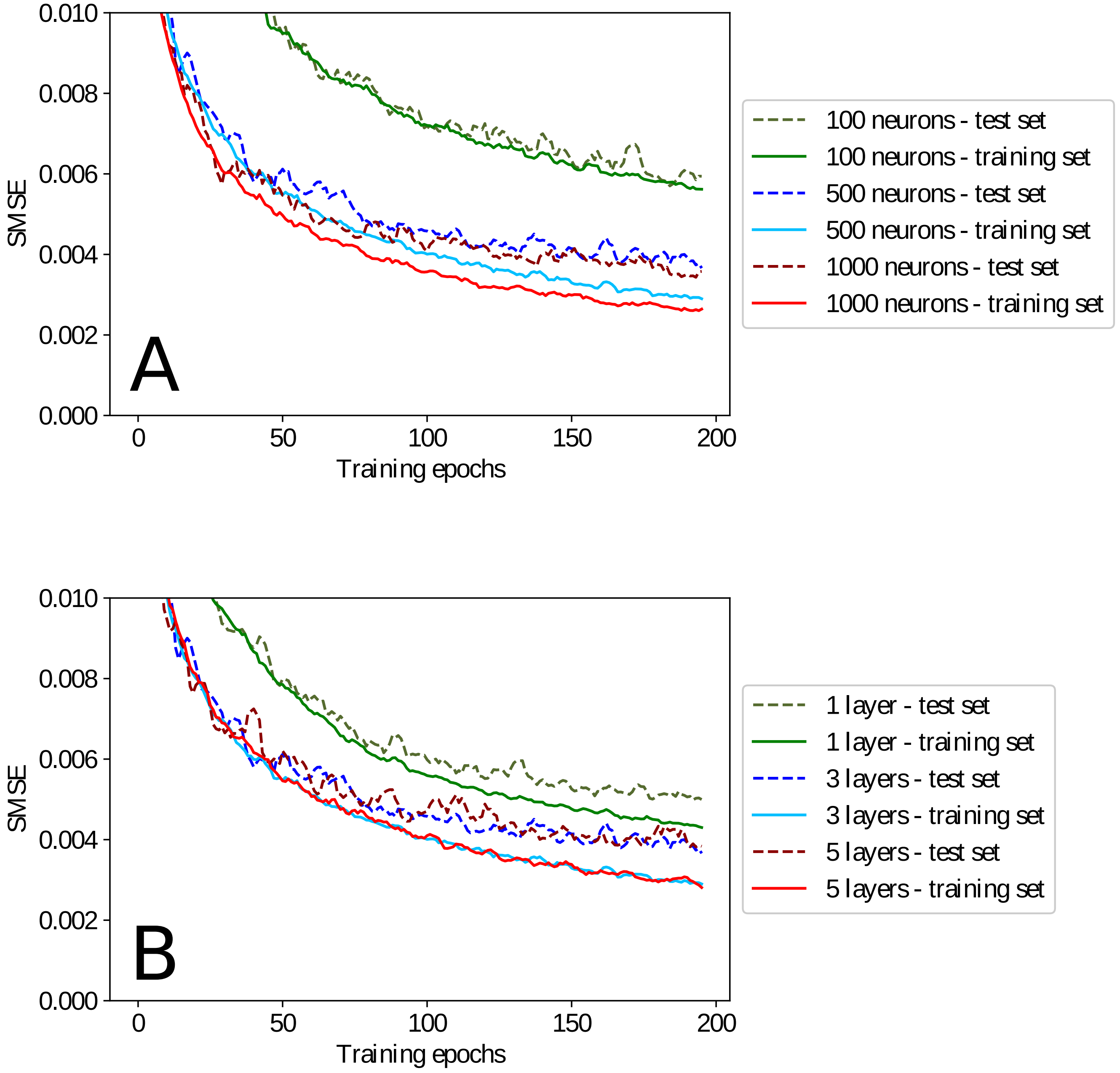

Fig. S4. Hyperparameter analysis of the RNN model over 200 epochs of training on case 1 database for (A) number of neurons in GRU cells and (B) number of stacked GRU layers.

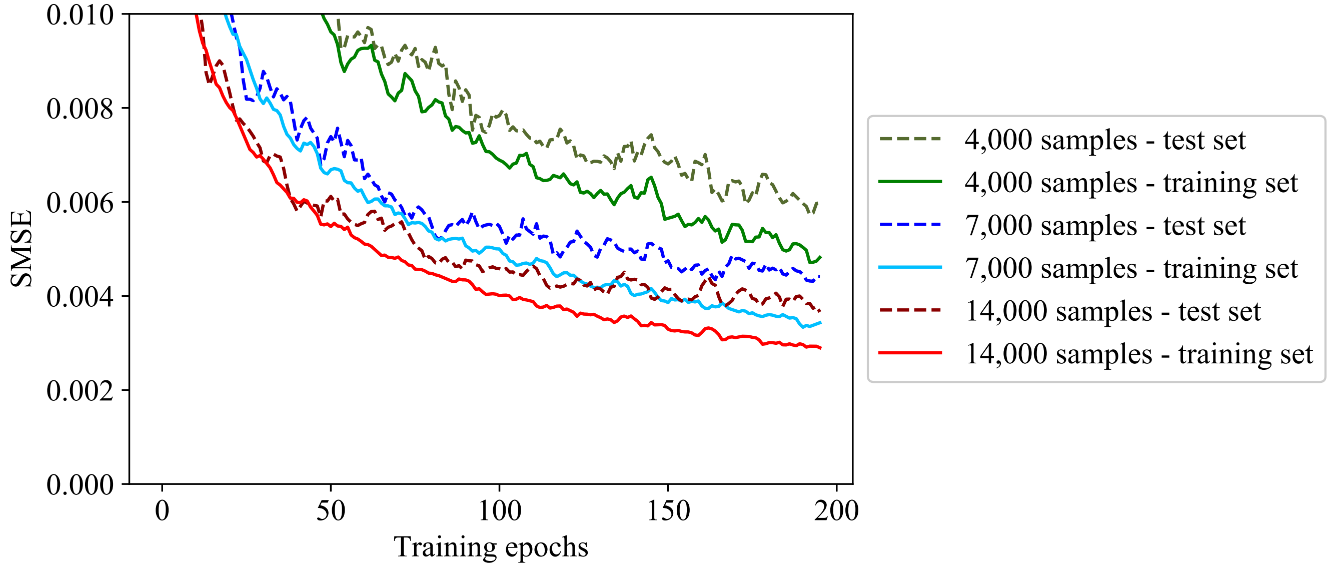

Fig. S5. Convergence test for the RNN over 200 epochs of training on case 1 database.

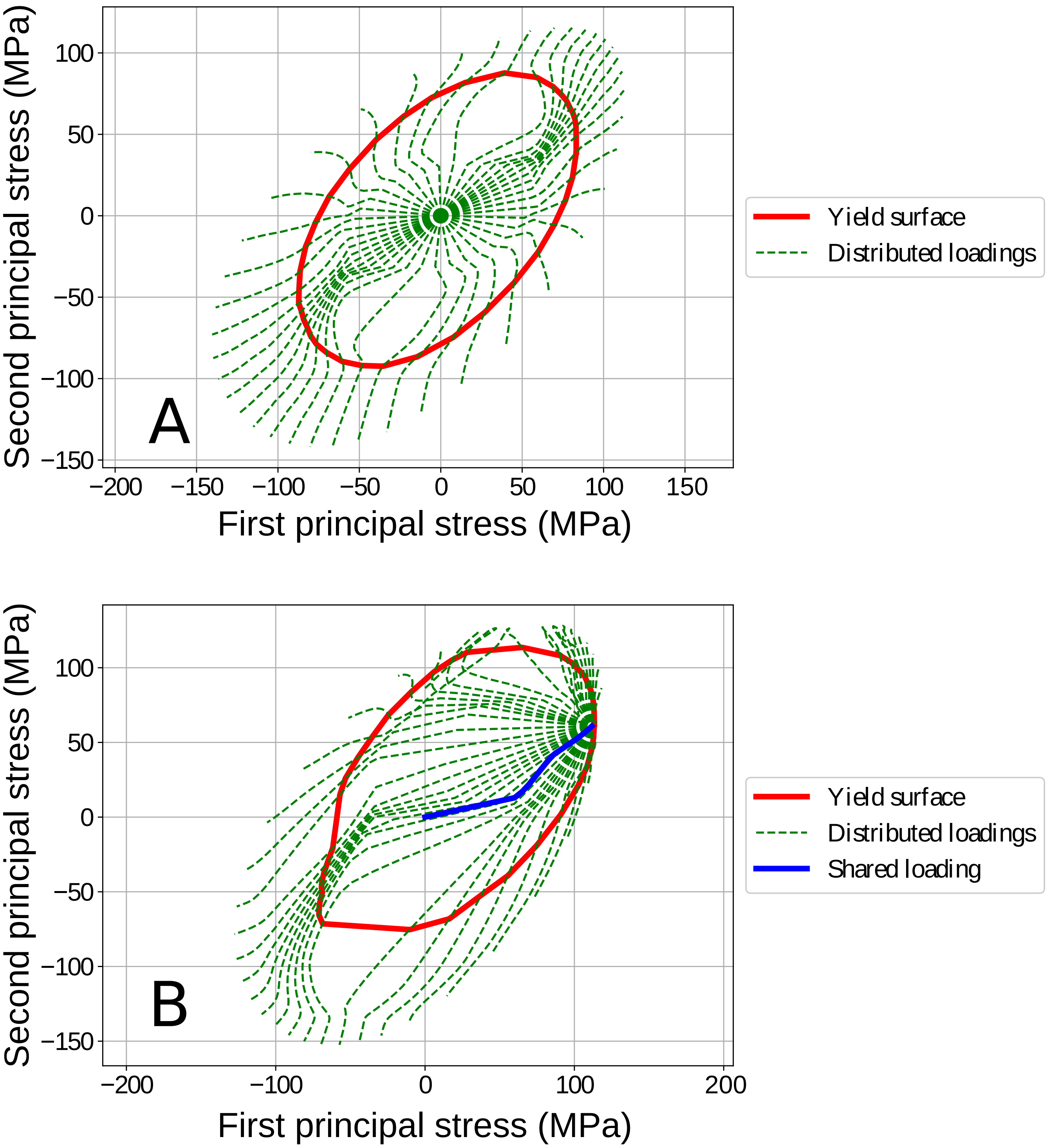

Fig. S6. Yield surface construction process for (A) original yield surface and (B) yield surface after loading.

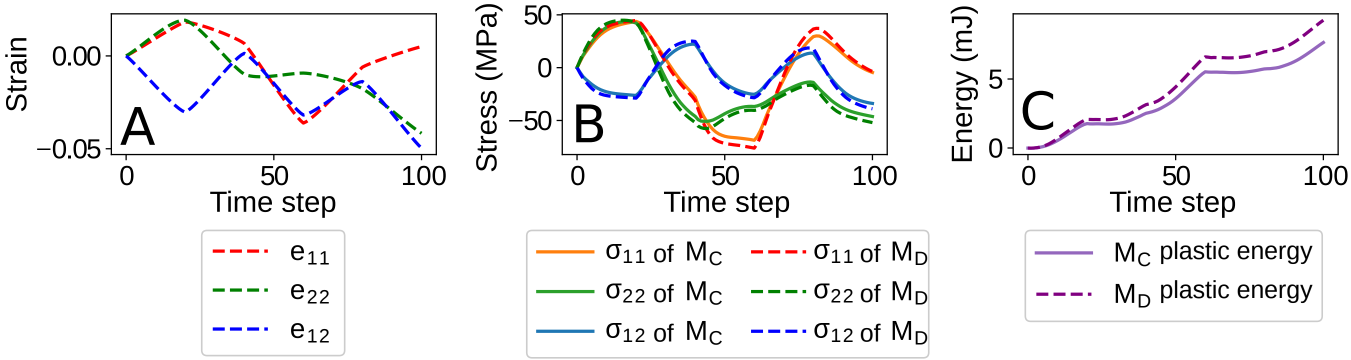

Fig. S7. Additional result for illustrative example 2. In this case considering two RVEs with different microstructures but undergoing the same loading history. The RVEs are labeled as $M_C \equiv [35\%, 8\ \mu\text{m}, 5\ \mu\text{m}]$ and $M_D \equiv [5\%, 6\ \mu\text{m}, 5\ \mu\text{m}]$ in order to refer to the microstructures C and D in Fig. S2, respectively.

Table S1. Parameter ranges for RVE reconstruction and load-path design for case 2.

	v (%)	r (μm)	c (μm)	$E = [e_{11},\, e_{22},\, e_{12}]$
Min	5	3	8	$[-0.02,\, -0.02,\, -0.02]$
Max	40	10	20	$[0.02,\, 0.02,\, 0.02]$

Table S2. Metrics comparison between trained RNN architectures after 500 epochs of training for case 2.

RNN architecture	Train set SMAE	Test set SMAE	Train set SMPED	Test set SMPED
Configuration A – hybrid mix	0.00557	0.00888	0.00233	0.00243
Configuration B – hidden state initialization	0.00345	0.00367	0.00130	0.00144
Configuration C – secondary hidden state	0.00242	0.00256	0.00104	0.00104

Table S3. Matrix and fiber material properties for case 1.

Matrix Young's Modulus (E_m)	68.9 GPa
Matrix Poisson's ratio (ν_m)	0.33
Matrix Voce isotropic hardening<br>$\sigma_{iso,m} = B - (B - A)\exp(-n\varepsilon)$	$A = 74.4$ MPa<br>$B = 144.98$ MPa<br>$n = 7.25$
Fiber density (ρ_f)	1.0 g/cm^3
Fiber Arruda shear coefficient (μ_f)	166 MPa
Fiber Arruda locking stretch (λ_f)	2.8
Fiber Arruda compressibility coefficient (D_f)	0.0025

Table S4. Matrix and fiber material properties for case 2.

Matrix Young's Modulus (E_m)	4.07 GPa
Matrix Poisson's ratio (ν_m)	0.34
Matrix Voce isotropic hardening<br>$\sigma_{iso,m} = B - (B - A)\exp -n\varepsilon$	$A = 16.44$ MPa<br>$B = 69.08$ MPa<br>$n = 102.5$
Matrix kinematic back-stresses ($n_{kin,m}$)	2
Fiber Young's Modulus (E_f)	15 GPa
Fiber Poisson's ratio (ν_f)	0.2

Table S5. Tabular values of the hardening law for the matrix.

strain	stress (MPa)
0	16.44623084
1.82E-04	19.0853206
3.49E-04	21.86697699
8.62E-04	24.69504529
0.00163815	27.43028185
0.00240308	30.20861866
0.00317509	33.0632108
0.00395132	36.08357382
0.00493514	39.04757864
0.00636065	41.87896426
0.00790903	44.82970433
0.00952196	47.81359834
0.01127912	50.84390993
0.01319586	53.77807359
0.01545202	56.71886509
0.01774464	59.87847969
0.02049382	62.86237823
0.02939382	65.87610888
0.04376	68.174796
0.0987	69.088644
1	70

References

1. Bessa M, et al. (2017) A framework for data-driven analysis of materials under uncertainty: Countering the curse of dimensionality. *Computer Methods in Applied Mechanics and Engineering* 320:633 – 667.
2. Karpathy A, Fei-Fei L (2015) Deep visual-semantic alignments for generating image descriptions in *The IEEE Conference on Computer Vision and Pattern Recognition (CVPR)*.
3. Vinyals O, Toshev A, Bengio S, Erhan D (2015) Show and tell: A neural image caption generator in *The IEEE Conference on Computer Vision and Pattern Recognition (CVPR)*.
